# PECAM1 plays a role in the pathogenesis and treatment of bone metastases

**DOI:** 10.3389/fgene.2023.1151651

**Published:** 2023-03-15

**Authors:** Zhuo-Tao Liang, Jia-Ke Li, Jiong Li, Hao Tang, Chao-Feng Guo, Hong-Qi Zhang

**Affiliations:** ^1^ Department of Spine Surgery and Orthopaedics, Xiangya Hospital, Central South University, Changsha, Hunan, China; ^2^ National Clinical Research Center for Geriatric Disorders, Xiangya Hospital, Central South University, Changsha, Hunan, China; ^3^ Department of General Surgery, The Third Xiangya Hospital of Central South University, Changsha, China

**Keywords:** PECAM1, bone metastasis, osteoclast, breast cancer, prostate cancer, biomarker

## Abstract

Bone is the third most common metastatic site for all primary tumors, the common primary focus of bone metastases include breast cancer, prostate cancer, and so on. And the median survival time of patients with bone metastases is only 2–3 years. Therefore, it is urgent to develop new targets to diagnose and treat bone metastases. Based on two data sets GSE146661 and GSE77930 associated with bone metastases, it was found that 209 genes differentially expressed in bone metastases group and control group. PECAM1 was selected as hub-gene for the follow-up research after constructing protein-protein interaction (PPI) network and enrichment analysis. Moreover, q-PCR analysis verified that the expression of PECAM1 decreased in bone metastatic tumor tissues. PECAM1 was believed to be possibly related to the function of osteoclasts, we knocked down the expression of PECAM1 with shRNA in lymphocytes extracted from bone marrow nailed blood. The results indicated that sh-PECAM1 treatment could promote osteoclast differentiation, and the sh-PECAM1-treated osteoclast culture medium could significantly promote the proliferation and migration of tumor cells. These results suggested that PECAM1 may be a potential biomarker for the diagnosis and treatment of bone metastases of tumor.

## Introduction

Cancer has been one of the major diseases causing death, with more than 20 million new cases worldwide every year (Sung et al., 2021). Its fatal reason lies that it can leave the primary site of the tumor, and spread to other parts of the body through a complicated process called metastases, and therefore causing the failure of organ function and eventually leading to death. Bone is the third most common site of metastases for all primary tumors (von Moos et al., 2019), and the common primary sites of bone metastases include breast cancer, prostate cancer, and thyroid cancer, in order. It has been reported that more than 70% of breast cancer patients and prostate cancer patients would eventually develop bone metastases (Boxer et al., 1989; Coleman, 2006). The median survival time of patients with bone metastases was only 2–3 years. Therefore, it is urgent to develop new targets to diagnose and treat bone metastases of tumor (Liang et al., 2020).

As a dynamic tissue, bone plays an important role in supporting structure and movement. It is composed of resident cells with different functions. Among these cells, osteoblasts and osteoclasts are the core, which maintain bone remodeling and absorption (Hofbauer et al., 2014; Sims and Martin, 2014). The physiological variations of bone are strictly regulated, but disease and aging will alter this environment, and provide suitable “soil” for the metastases of a variety of primary tumors. Nevertheless, the specific mechanism of bone metastases still remains to be clarified. Currently, it is believed that the site of bone metastases has abundant circulation of blood. The presence of bone trabeculae, and the alteration of the intracellular microenvironment of bone may also cause the development of bone metastases (Yip et al., 2021). The alteration of microenvironment of cells in bone has always been the research focus on tumor bone metastases.

The most typical example of tumor cells altering the bone environment is the concept of “vicious cycle” proposed by Muddy (Mundy, 1997). This is the first time to point out that tumor cells can stimulate bone cells to secrete various growth factors such as TGF-β and IGF, thus promoting tumor cell colonization and further bone destruction. Herein, osteoclasts act as a major role (Chen et al., 2010). Osteoclasts are large multinucleated syncytial cells formed by the fusion of bone marrow-derived mononuclear cells. Mature osteoclasts attach to the bone surface through actin rings and secrete a variety of proteases to demineralize the bone matrix and degrade proteins to form cavities (Buijs et al., 2011; Suva et al., 2011). While the RANKL/OPG pathway is the key pathway in osteoclast differentiation process, nuclear factor k-B ligand (RANKL) activates NF-κβ to stimulate osteoclast differentiation by combining with RANK, OPG can competitively inhibit this process by combining with RANKL (Kearns et al., 2008). In addition, some factors can also directly induce osteoclast activation, or stimulate adjacent cells to produce RANKL activation, including interleukin-6 (IL-6) (Weilbaecher et al., 2011), IL-11 (Kang et al., 2003), and soluble intercellular adhesion molecule 1 (ICAM-1) (Ell et al., 2013). Some studies demonstrated that tumor cells could directly secrete these factors, thereby activating osteoclast differentiation. Because of the understandings, bisphosphonates have been applied, such as zoledronic acid and denosumab could treat patients with bone metastases or improve their quality of life by targeting osteoclasts to limit bone turnover (D'Oronzo et al., 2019). However, the role of osteoclasts in bone metastases is far from fully understood.

In this study, we selected GSE146661 and GSE77930 data sets from the Gene Expression Omnibus (GEO) database, which were sequencing items related to bone metastases of breast cancer and prostate cancer (the two most common primary lesions of bone metastases cancer). Moreover, various bioinformatics analyses were also performed, so as to explore the gene expression and protein-protein interactions during tumor bone metastases, and to establish new biomarkers for its diagnosis and treatment.

## Methods and materials

### Dataset inclusion

Gene expression profile data (GSE146661 and GSE77930) were downloaded from GEO (Supplementary Tables S1, S2). Inclusion criteria of gene expression data: 1) the samples used for analysis were the tissues; 2) the experimental group was tumor bone metastases tissues, and the control group was primary focus tissue or other metastatic tissues, and the samples with combined bone metastases should be excluded; 3) complete information could be obtained for analysis; 4) the sample size of each study set was greater than 10. The array data of GSE146661 included 7 cases of breast cancer with bone metastases and 4 cases of primary lesion tissues as the control group, and the array data of GSE77930 included 11 prostate cancer with bone metastases and 15 other prostate cancer metastases as the control group.

### Subjects

We recruited five patients with bone metastases and 7 seven patients without tumors as controls (Supplementary Table S3). The inclusion criterion used for the patients are diagnosed by imaging examination which have primary tumor and bone metastases. The inclusion criteria for the control group were sex-matched patients without tumor, including healthy individuals and patients with bone fracture at our medical examination center. The patients in the control group were subjected to a comprehensive examination to confirm that no bone metastases existed.

This study was approved by the Ethics Committee of Xiangya Hospital of Central South University (Reference: 201703358). Informed written consent was given by all the subjects and their legal guardians prior to participation in the study.

### Differential gene analysis

Differentially expressed genes (DEGs) between tumor bone metastases samples and control group samples were analyzed using GEO2R (http://www.ncbi.nlm.nih.gov/geo/geo2r). GEO2R is a network tool that can compare and analyze DEGs in samples through GEOquery R packages of Bioconductor project. The adjusted *p*-values and |log2-fold variation| (|log2FC|) values were employed to assess the significance of DEGs, with the threshold set to |log2FC| > 1 and the adjusted *p*-values <0.05. Gene ontology (GO) is commonly used for comprehensive evaluation of gene function. Kyoto Encyclopedia of Genes and Genomes (KEGG), a database that annotates the advanced functions of biological systems at the molecular level. Enrichment of DEGs was analyzed and visualized by GOplot package (version 1.0.2) and ggplot2 (version 3.3.3) of R (https://www.r-project.org). The truncation criterion was set to *p*-value less than 0.01.

The protein-protein interaction (PPI) network of DEGs was constructed using the STRING (http://string-db.org/) database (which searched for interacting genes and provided important information about protein-protein interactions (PPI)), and the PPI network was processed and analyzed using Cytoscape (version 3.7.2). The threshold criterion was a composite score≥0.9. The truncation standard was set to *p*-value less than 0.05.

### Quantitative real-time polymerase chain reaction (qPCR)

During the operation, 4 cases of bone metastases and 4 cases of control bone tissues without tumor were collected. Informed written consent was given by all the subjects and their legal guardians prior to participation in the study. This study was approved by the Ethics Committee of Xiangya Hospital of Central South University (Reference: 201703358).

Total RNA were extracted from the organization. The experimental method used for qRT-PCR was based on a previously described method (Liang et al., 2019). The primers used in this study were listed in Table 1.

**TABLE 1:** Primers for quantitative real-time PCR (qPCR).

Gene		Primer sequences (5′to 3′)
PECAM1	R	ATG​GAG​CAG​GAC​AGG​TTC​AGT​C
	F	AAG​TGG​AGT​CCA​GCC​GCA​TAT​C
COL3A1	R	TGT​GTT​TCG​TGC​AAC​CAT​CC
	F	CTT​CTC​TCC​AGC​CGA​GCT​TC
CD4	R	GAC​GGC​AGC​CTG​ACA​GTA​ATG​AG
	F	CAC​AGC​GCA​GGT​GTA​CTC​GCC​C
ITGB3	R	GAAGGTAGACGT GGCCTCTTT
	F	CGC​TAA​ATT​TGA​GGA​AGA​ACG
TLR4	R	CGA​TGG​GAA​CAT​TCA​GGG​CAG​AG
	F	ACA​CAT​TCA​TGG​AGG​CAC​TGG​AAC
RN18s	R	AGA​AAC​GGC​TAC​CAC​ATC​CA
	F	CCC​TCC​AAT​GGA​TCC​TCG​TT

### Western blotting (WB)

The experimental method used for WB was based on a previously described method (Liang et al., 2019). The following primary antibodies were used: GAPDH (1:5,000; CST, United States), PECAM1 (1:500; Abcam, United States), RANKL (1:500; Abcam, United States), OPG (1:800; CST, United States). The membranes were subsequently incubated with secondary antibodies (1:10,000, Proteintech, China) at room temperature for 1 h. The results were then detected using the Chemiluminescent Protein Detection Module (Thermo Scientific, United States).

### Immunofluorescence (IF)

Cells were placed on slides and fixed with 4% paraformaldehyde for 15 min, incubated with 0.3% triton solution for 10 min, sealed with 5% BSA for 30 min and then incubated with Ki67 (1:500; CST, United States) primary antibody at 4°C overnight. The following morning, the cells were incubated with a fluorescence secondary antibody (1:300, Abcam, United States) at room temperature for 1 h. Images were then acquired by a confocal microscope.

### shRNA transfection

First, the cells were seeded into 24-well plates at a density of 1 × 10^4^ cells per well. The cells were then transfected with shRNAs using riboFECTTM CP reagent (RiboBio, China) at final shRNA concentrations of 50 nM in accordance with the manufacturer’s recommended protocols. The shRNA sequences used to knock-down PECAM1 were: sense 5′-GAA​UUC​UCG​AGA​CCA​GAA​UUU and antisense 5′-AUU​CUG​GUC​UCG​AGA​AUU​CUU. Forty-eight hours after transfection, the expression levels of PECAM1 protein were verified by Western blotting (WB).

### Scratch assay

HCT116 and SW620 cells were seeded at a density of 1 × 105 cells/well in 24-well plates until they were 90% confluent. Scrape wounds were generated using a 20-µL pipette tip; then, cells were cultured with a serum-free medium for 48 h. Wound closure was monitored and photographed at 0, 24, and 48 h using microscope.

### Trap staining

The cells were fixed with 4% paraformaldehyde for 15 min, then 500 uL of sodium nitrite and parafuchsin hydrochloride were mixed evenly, 18 mL of sodium acetate buffer solution was added, and then 1 mL of naphthol AS-BI phosphate solution was added. Afterwards, 0.282 g of potassium sodium tartrate was weighed and finally added, fully dissolved to prepare the working solution. Then the working solution was added dropwise onto the cell climbing sheets. The sheets were incubated at 37°C for 1–2 h, and then washed with distilled water for three times. Subsequently, the cells were stained with hematoxylin staining solution for 1 min, washed with tap water, differentiated with differentiation solution, washed with tap water. And finally the experimental results were observed by microscopy.

### Statistical analysis

All data are expressed as mean ± standard deviation (SD). The student’s t-test (unpaired or paired) was used to determine the significance of differences between the two groups. Analysis of variance (ANOVA) was used to determine the significance of differences between multiple groups. *p* < 0.05 was considered statistically significant.

## Results

### Identification of differentially expressed genes in bone metastases

**FIGURE 1:**
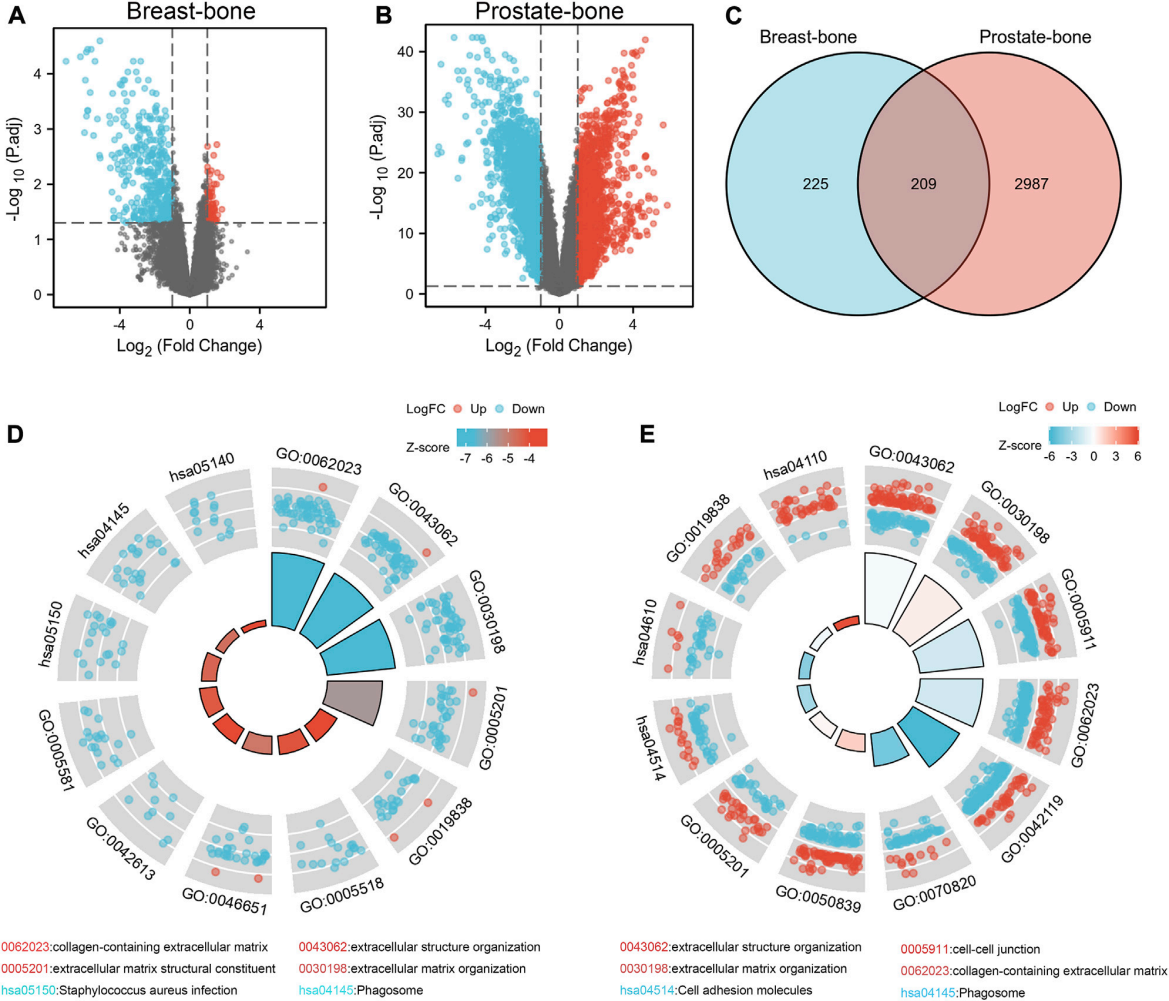
Identification and enrichment analysis of DEGs in bone metastases. The volcano map showed 434 DEGs in GSE146661 **(A)** and 3,196 DEGs in GSE77930 **(B)**. 209 DEGs were identified from GSE146661 and GSE77930 **(C)**. GO/KEGG joint enrichment analysis on GSE146661 and GSE77930 indicated that DEGs from GSE146661 and GSE77930 were mainly enriched in extracellular structure organization; extracellular matrix organization; cell-cell junction; cell adhesion and collagen-containing extracellular matrix plate pathways **(D, E)**.

According to the GEO2R analysis, differentially expressed genes (434 in GSE146661 (Supplementary Table S1), 3,196 in GSE77930 (Supplementary Table S2)) were identified after normalization of microarray data (Figures 1A, B). Venn diagram analysis of bioinformatics and evolutionary genomics platforms indicated that a total of 209 common DEGs (cDEGs) were included in the two datasets (Figure 1C; Supplementary Table S4).

We performed GO/KEGG joint enrichment analysis on GSE146661 and GSE77930 respectively (Supplementary Tables S5, S6). The analysis results indicated that DEGs from GSE146661 and GSE77930 were mainly enriched in extracellular structure organization and extracellular matrix organization in biological process (BP) analysis. In addition, the analysis of cell composition (CC) illustrated that the DEGS of GSE146661 and GSE77930 were mainly enriched in cell-cell junction and collagen-containing extracellular matrix plate. Molecular function (MF) analysis indicated that the DEGS of GSE77930 was mainly enriched in cell adhesion molecule binding and extraceller matrix structural constituent plate. The DEGS of GSE146661 was mainly enriched in the extracellular matrix structural constituent and growth factor binding plate. And KEGG pathway analysis suggested that most of the DEGs in GSE77930 were mainly involved in cell adhesion molecules and complement and coagulation cascades pathway, while most of the DEGs in GSE146661 were mainly involved in *staphylococcus aureus* infection and phagosome pathway (Figures 1D, E). Herein, we found a lot of overlapping parts in the enrichment analysis results of the two gene sets.

### Analysis of selected differential expressed genes

**FIGURE 2:**
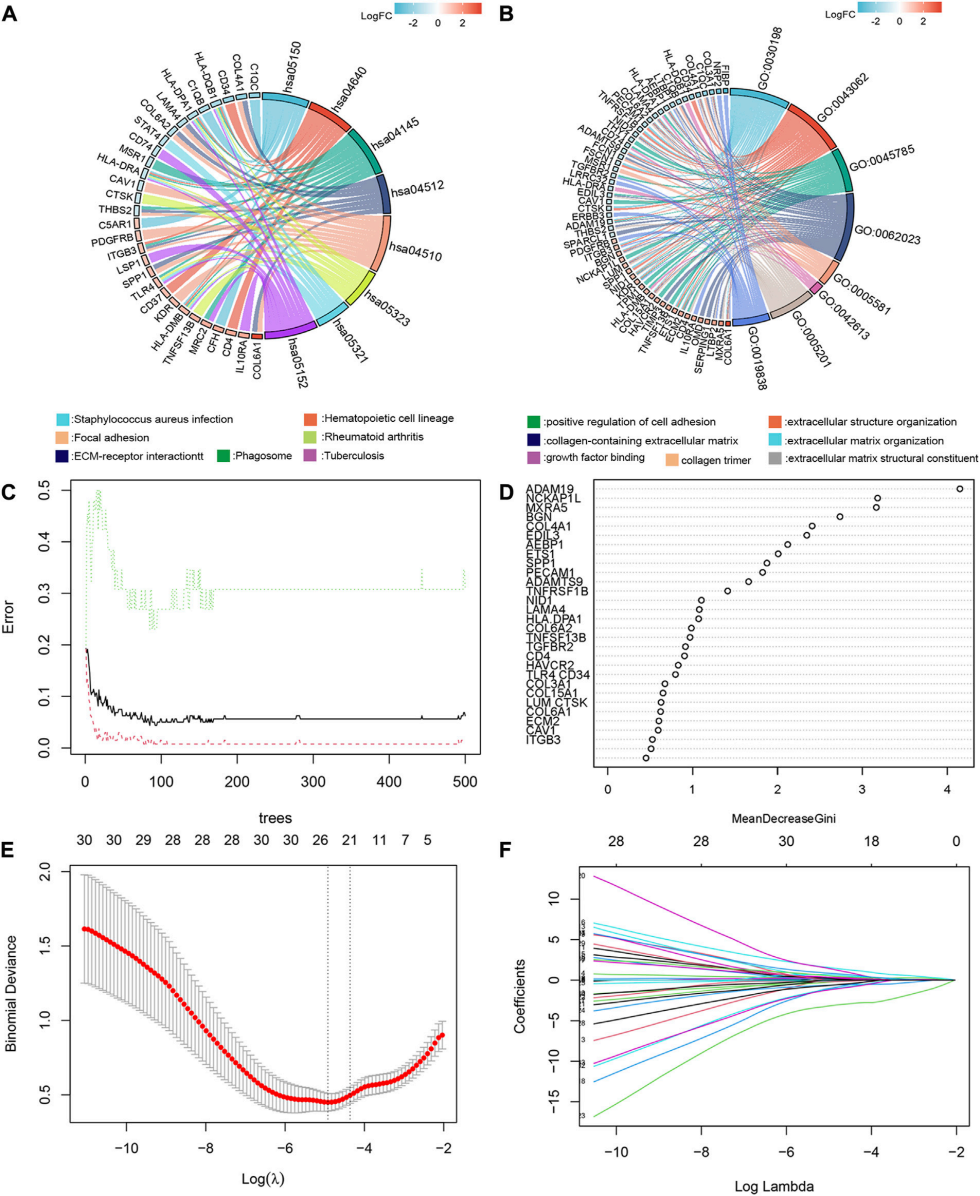
GO/KEGG enrichment analysis, random forest algorithm and LASSO regression modeling to screen key gene variables capable of distinguishing bone metastases and controls. KEGG **(A)** and GO **(B)** enrichment analysis of 209cDEGs. **(C)** The out-of-bag (OOB) error rate reached a minimum when the number of trees was equal to 85. **(D)** 28 of 36 genes were selected as important variables *via* random forest algorithm. **(E)** The optimal lambda was determined when the the model reached an optimum. **(F)** LASSO coefficient profiles of the candidate genes for bone metastases.

And therefore, further KEGG (Figure 2A) and GO (Figure 2B) enrichment analysis of 209 common differentially expressed genes (cDEGs) in the two gene sets was conducted. We attached importance to genes of encoding secretory proteins and cell membrane proteins, which usually played a key role in tumor-bone microenvironment interactions, such as the pathways related to extracellular matrix formation and cell adhesion. We listed these 36 candidate genes in Supplementary Table S7. We also employed a random forest algorithm to screen key gene variables capable of distinguishing bone metastases and controls, with a relative importance greater than 0.5 as the filtering criterion. The out-of-bag (OOB) error rate reached a minimum when the number of trees was equal to 85 (Figure 2C), and 28 of 36 genes were selected as important variables (Figure 2D). Based on the fivefold cross-validation, the model reached an optimum when lambda was equal to 0.007, containing 21 key gene variables (Figures 2E, F).

### Protein-protein interaction network construction and analysis of hub-genes

**FIGURE 3:**
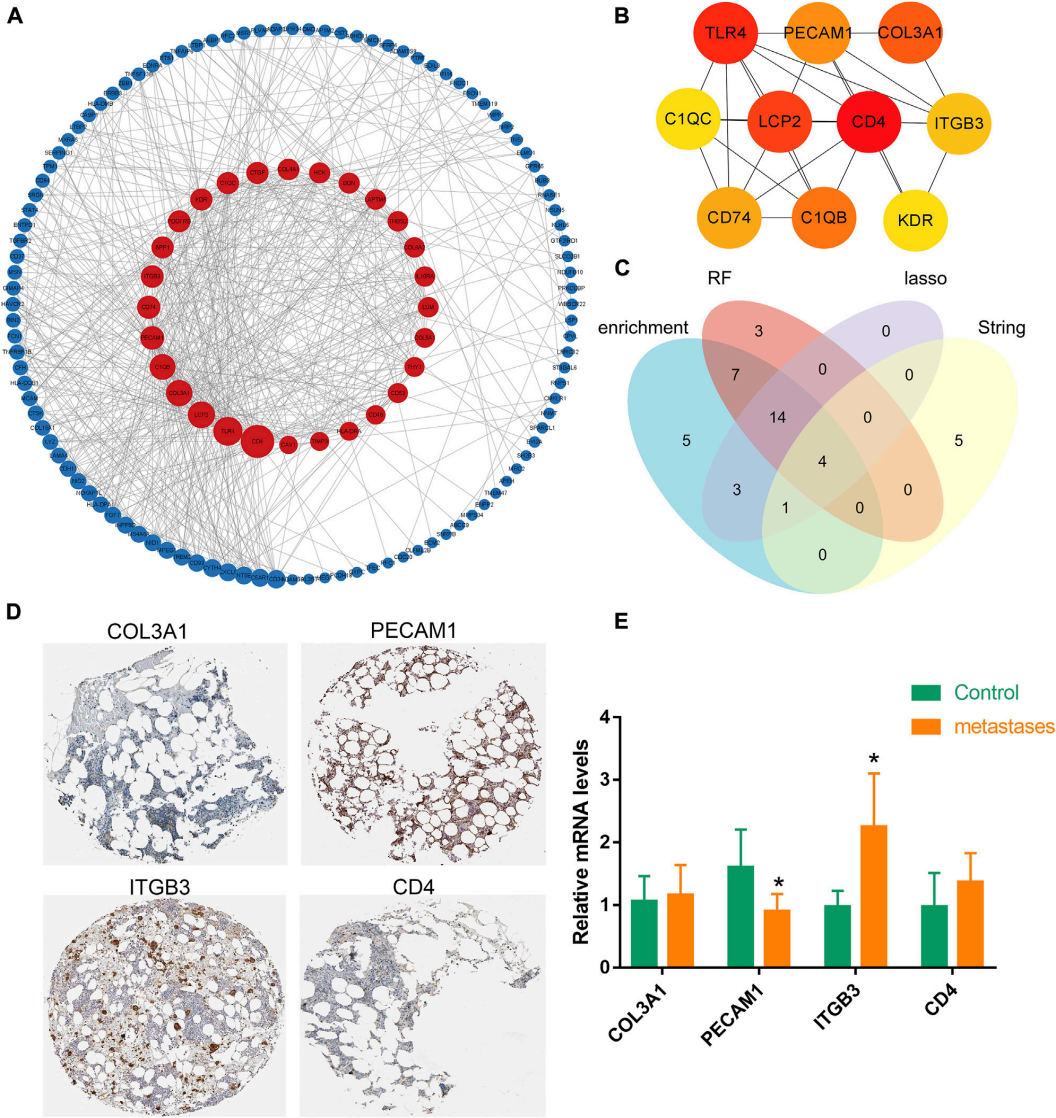
Expression of hub-genes in bone. **(A)** A PPI network was generated based on cDEGs using Cytoscape (combined score ≥ 0.9), upregulated genes are marked in dark red; downregulated genes are marked in dark blue. **(B)** The interaction network of the 10 nodes with the highest degree scores. The red, orange, and yellow nodes represented the top 10 hub genes in the network. **(C)** Venn diagram was mapped by the screened gene in PPI and the candidate genes selected by random forest algorithm and LASSO regression modeling, and four overlapped genes were obtained. **(D)** Four hub-genes in bone marrow tissues were verified by THPA database. It was showed high level of PECAM1 and ITGB3 genes in bone marrow, while COL3A1 and CD4 exhibited low level. **(E)** q-PCR results indicated that the expression levels of PECAM1 decreased significantly, but COL3A1; ITGB3 and CD4 had no significant difference in the expression of bone metastases tumor tissues. Data are shown as the mean ± SD, * means *p* <0.05 vs. the control group.

Next, a PPI network was constructed for 209 differential genes obtained in Figure 1C. The PPI network consisted of 136 nodes and 602 edges (Figure 3A). The top ten ranked hub-gene was selected for subsequent in-depth analysis based on the degree value (an indicator representing the importance of each node) as the selection condition (Figure 3B; Supplementary Table S7). After that, Venn diagram was mapped by the screened gene in PPI and the candidate genes selected from enrichment analysis, and four overlapped genes were obtained, namely, Collagen Type III Alpha 1 (COL3A1), platelet/endothelial cell adhesion molecule-1 (PECAM1), β3 integrin (ITGB3), CD4 (Figure 3C). Then, the correlation between these four hub-genes in bone marrow tissue and different cells were further verified by THPA database. It was showed high level of PECAM1 and ITGB3 genes in bone marrow, while COL3A1 and CD4 exhibited low level (Figure 3D). Mononuclear macrophages in bone marrow cavity were the precursor cells of osteoclasts, which could differentiate into osteoclasts after being activated. Meanwhile, we found the list of macrophage-specific marker genes through THPA database. It was demonstrated that PECAM1, CD4 and COL3A1 had certain cell specificity, and they could be used as marker genes of macrophages (Supplementary Figure S1). The mRNA expression levels of these genes were verified in human primary bone metastases tumor tissues and normal bone tissues by q-PCR, and the results indicated that the expression levels of PECAM1 decreased and ITGB3 increased significantly, but COL3A1 and CD4 had no significant difference in the expression of bone metastases tissues (Figure 3E). Eventually, PECAM1 gene was selected for further analysis.

### Knocking down PECAM1 expression promotes osteoclast differentiation

**FIGURE 4:**
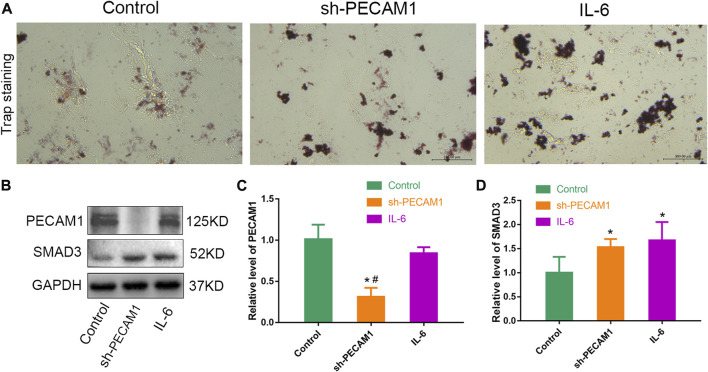
Knocking down PECAM1 expression promotes osteoclast differentiation. **(A)** Representative images of trap staining in either control group, sh-PECAM1 treatment group and IL-6 treatment mononuclear macrophages group. **(B–D)** Protein levels of PECAM1 and SMAD3 in either control group, sh-PECAM1 treatment group and IL-6 treatment mononuclear macrophages group. Scale bar, 100 μm. Data are shown as the mean ± SD, * means *p* < 0.05 vs the control group. # means *p* < 0.05 vs the IL-6 treated group.

In view of the central role of osteoclast in the colonization and proliferation of tumor cells in bone tissue, it is meaningful to explore whether PECAM1 can affect the osteoclast differentiation of mononuclear macrophages. We therefore obtained monocyte macrophages from the nailed blood of human bone marrow, and induced osteoclast differentiation of mononuclear macrophages by using osteoclast differentiation induction medium. We set up three groups, namely, control group, sh-PECAM1 treatment group and IL-6 treatment mononuclear macrophages group. Trap staining results revealed that compared with CT group, knockdown of PECAM1 expression would promote osteoclast differentiation of mononuclear macrophages, and the result was close to that of IL-6 treatment group (Figure 4A). Meanwhile, we also collected the total protein of mononuclear macrophages after 7 days of induced differentiation. The WB test results indicated that the expression of PECAM1 protein significantly decreased in the sh-PECAM1 group, as compared to the other two groups (Figures 4B, C). In addition, the value of the key protein smad3 in the osteoclast differentiation pathway significantly increased in the sh-PECAM1 and IL-6 groups, suggesting that the osteoclast differentiation pathway was activated (Figures 4B, D).

### sh-PECAM1 treatment of osteoclast conditioned medium could promote tumor cell proliferation and migration

**FIGURE 5:**
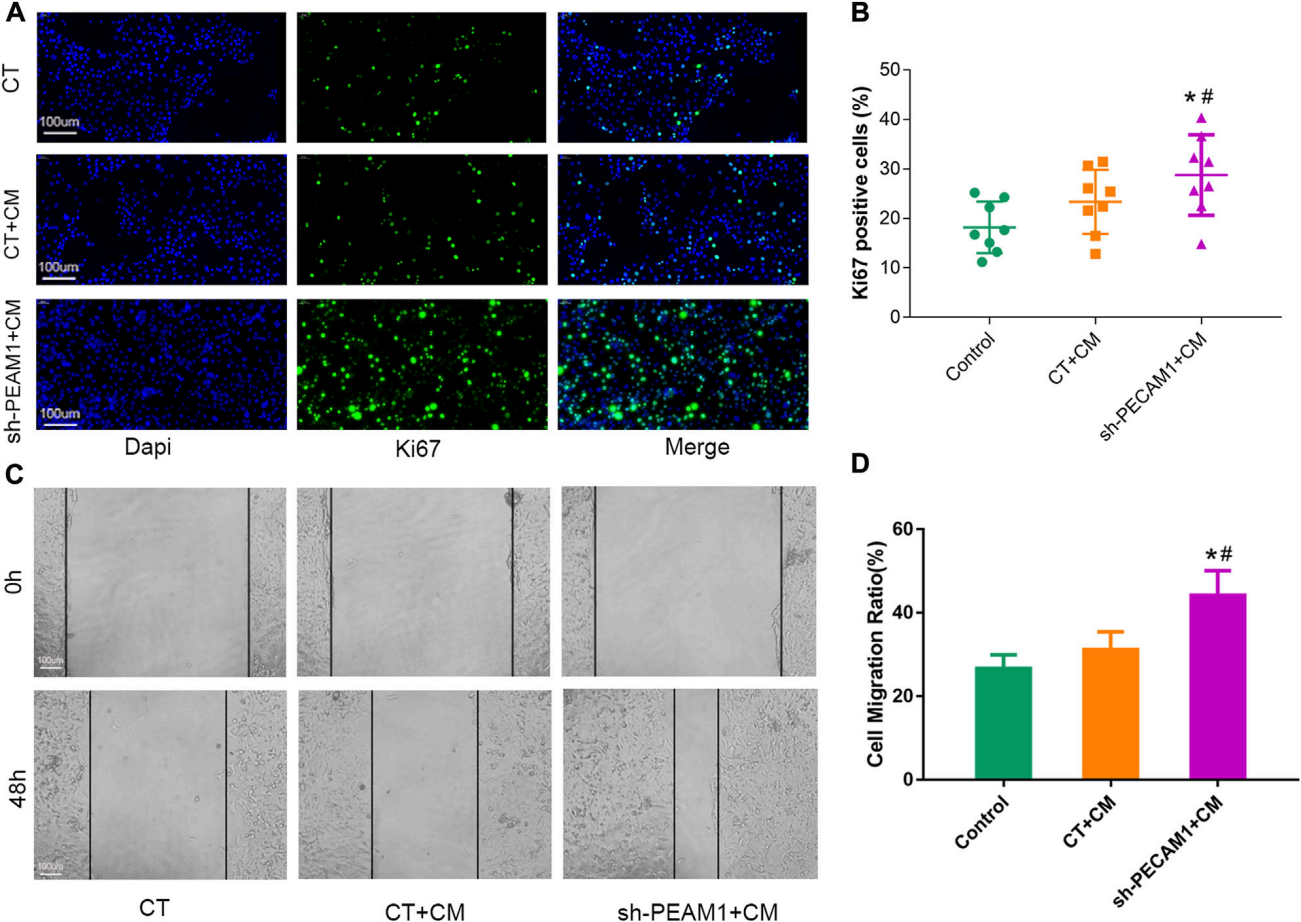
sh-PECAM1 treatment of osteoclast conditioned medium could promote tumor cell proliferation and migration. **(A, B)** Representative images of Ki67 immunofluorescence staining 627 in either control; treatment group with untreated osteoclast conditioned medium (CT + CM) and sh-PECAM1 treated osteoclast conditioned medium group (sh-PECAM1+CM). Scale bar, 100 μm. **(C, D)** Representative images of scratch assay in either control; CT + CM and sh-PECAM1+CM group. Scale bar, 100 μm. Data are shown as the mean ± SD, * means *p* < 0.05 vs the control group. # means *p* < 0.05 vs the CT + CM treated group.

MCF-7 cells (breast cancer cell line) were cultured using different treatments of osteolast conditioned medium to observe their effects on tumor cell proliferation and migration ability. We set up three different subgroups, *viz.* control group without any special treatment (CT), treatment group with untreated osteoclast conditioned medium (CT + CM), sh-PECAM1 treated osteoclast conditioned medium group (sh-PECAM1+CM). Ki67 staining results illustrated that the proliferative capacity of MCF-7 cells in the sh-PECAM1+CM group was significantly higher than that of the other two groups (Figures 5A, B). Moreover, the scratch assay showed that the migration ability of MCF-7 cells in sh-PECAM1+CM group was stronger than that of the other two groups (Figures 5C, D). The above results suggested that sh-PECAM1-treated osteoclast conditioned medium would stimulate the proliferation and migration of tumor cells, therefore creating a more favorable situation for bone metastases of tumors.

## Discussion

Bone is one of the most common metastases sites among various tumors, for instance, the most common distant metastases of breast cancer and prostate cancer is bone (Bray et al., 2018). So far, the mechanism of bone metastases of primary tumor remains unclear. Nevertheless, multiple genes and pathways have been confirmed to participate in this process, making the research of bone metastases mechanism be extremely sophisticated. Therefore, it is urgent to improve our understanding of the mechanism of tumor bone metastases. What’s more, employing genomics, transcriptomics, proteomics, and metabonomics analysis will be beneficial to develop better diagnosis and treatment strategies based on the identification of new biomarkers (Li et al., 2021).

In the present study, the RNA sequencing studies of prostate cancer and breast cancer bone metastases tissue samples were included for further analysis. Taking the bone metastases tissue as the experimental group, and primary tumor or other metastases sites as the control group, 209 common differentially expressed genes were selected through GEO2R analysis. It was found that these genes were mainly enriched in extracellular matrix formation, cell adhesion, growth factor binding and other pathways. We focused on some pathways encoding secretory proteins or cell membrane related proteins, because these genes may be involved in intercellular signal transduction. We also constructed a PPI network to further screen the hub-gene. Finally, we obtained four key candidate genes: ITGB3, PECAM1, CD4, and COL3A1. q-PCR results illustrated that the expression of PECAM1 in bone metastases was significantly lower than that of the normal bone tissues and ITGB3 level in bone metastases was significantly higher, while the other two genes had no significant difference. Moreover, based on THPA database analysis, we found that PECAM1 exhibited a higher cell specificity and was one of the marker genes of macrophages (Jackson, 2003). It has been known that osteoclasts were derived from the bone marrow monocyte-macrophage system. Previous works reported that knockdown of PECAM1 expression in mononuclear cells could lead to the enhanced osteoclast differentiation (Wu et al., 2009), which suggested that PECAM1 might regulate bone metastases of tumor by affecting osteoclast differentiation. Tumor bone metastases usually occurred first in the hypervascular axial bone containing red bone marrow, indicating that the slow blood flow in these areas could support the attachment of metastatic tumor cells to the surface of the endosteal membrane (Paget, 1989). Alternatively, anatomical features could not adequately explain the mechanism of metastases. The molecular characteristics of malignant cells (i.e., seeds) and their interaction with the intraosseous microenvironment (soil) played a more important role in promoting tumor metastases and diffusion (Brylka and Schinke, 2019).

Many studies demonstrated that cancer cells stimulated the generation of osteoclasts. In this theory, osteoclasts were often regarded as “bone digesters.” The secondary effects of bone resorption, including the physical space of tumor growth and the degradation of bone matrix to release prototype factors, promote tumor colonization in bone. The histological manifestations of bone metastases could be classified as osteolytic metastases, osteogenic metastases, and mixed metastases, which were based on the variations in osteolysis or sclerosis. Focal bone destruction occurred when osteoclast mediated bone resorption was dominant, resulting in what was described as “punched out” lytic lesions. In contrast, in bone metastases characterized by increased osteoblast activity, the metastatic bone exhibited dense osteosclerotic lesions (Thomas et al., 1999; Yin et al., 2003). Although these phenotypes represented both ends of the spectrum, autopsy studies indicated that the bone metastases in individual patients may be heterogeneous, that was, osteolytic at one site and osteogenic or mixed in another. In fact, most solid tumors metastasized to bone were mixed metastases, such as breast cancer and prostate cancer. This was because after the increase of osteoclast production, it would also release some growth factors, such as osteoblasts deposit growth factors, which promoted the formation of osteoblasts. From the above studies, it could be deduced that osteoblasts undoubtedly play a crucial role in the process of bone metastases of tumors (Nelson et al., 1995; Liu et al., 2004). But the specific mechanisms that mediated tumor bone metastases remain to be further investigated.

In the current status, there are several main directions regarding the regulation of tumor bone metastases by osteoclasts. One is that osteoclasts metabolize bone matrix to release a large amount of calcium ions. Calcium has been proved to support the growth of cancer cells in bone by activating calcium-sensitive receptors on the surface of tumor cells (Roudier et al., 2004). In addition to calcium, bone is also a storehouse of growth factors. Many factors that can promote tumor growth can be released and activated by osteoclasts, such as transforming growth factor-β(TGFβ). The release of these factors would create a vicious cycle, in which, enhanced tumor growth promotes osteolysis, and release additional growth factors, and therefore further aggravating tumor progression (Joeckel et al., 2014). Besides, the immune system in the bone marrow would also inhibit or promote the growth of a variety of cells, which would also affect tumor metastases to bone. It has been known that osteoclasts were derived from monocytes, and RANKL, the most important factor regulating osteoclast function, could be secreted not only by osteocytes, lymphocytes and macrophages (Xiong et al., 2011; Xiong et al., 2015; Brylka and Schinke, 2019). Furthermore, some factors that regulated the homing of immune cells to the action site cold also regulate the colonization of tumor cells (Saxena et al., 2021). Therefore, the regulation of osteoclasts on tumor bone metastases was investigated in the presented work. After knocking down the expression of PECAM1 in human monocytes, it could be found that PECAM1 could promote osteoclast differentiation. And RANKL/OPG, the pathway that stimulated osteoclast formation, was also activated, indicating that PECAM1 may promote osteoclast differentiation by activating the secretion of RANKL. Moreover, the research on the function of osteoclasts on tumor cells is also worth paying attention to. We collected the culture media of osteoclasts after 1 day of culture to treat MCF-7 cells, to investigate the effect of osteoclast culture medium on tumor cells. The results indicated that the osteoclast-conditioned medium could promote the proliferation of MCF-7 cells to a certain extent. Furthermore, it has been found that the osteoclast medium after sh-PECAM1 intervention significantly improved the proliferation and migration ability of tumor cells, and there was a significant difference when compared with the blank control group or the osteoclast media group without special treatment. The results indicated that PECAM1 gene might play a role in promoting osteoclast differentiation and tumor cell proliferation and migration in bone, which provided a new entry point for the diagnosis and treatment of bone metastases of tumor.

There are also some limitations in our study. For example, the gene set and sample size included in the bioinformatics analysis weren’t large. This may be because it is difficult to collect tissues including the primary site, as well as bone metastases and other metastases, resulting in fewer sequencing researches in this field. In the future, we should pay attention to the research trends in this field. And moreover, our own sequencing projects should be carried out to expand and verify the results. In addition, the verification experiment of candidate gene function needs to be further improved to better understand the regulation mode of PECAM1 in the process of tumor bone metastases. Moreover, other genes such as TLR4, CD4, COL3A1, and ITGB3 are also of great research value, and whether other genes are also involved in the regulation of tumor bone metastases will be investigated in the later studies.

## Data Availability

The datasets presented in this study can be found in online repositories. The names of the repository/repositories and accession number(s) can be found in the article/Supplementary Material.
