# Supplementary material for: PECAM1 plays a role in the pathogenesis and treatment of bone metastases
Source: Front Genet. 2023 Mar 15;14:1151651. doi: 10.3389/fgene.2023.1151651 (PMC10050551; doi:10.3389/fgene.2023.1151651)
Supplement: Supplementary file 1 [file Presentation1.zip › supplementary/supplementary figure.docx]

**Figure S1**


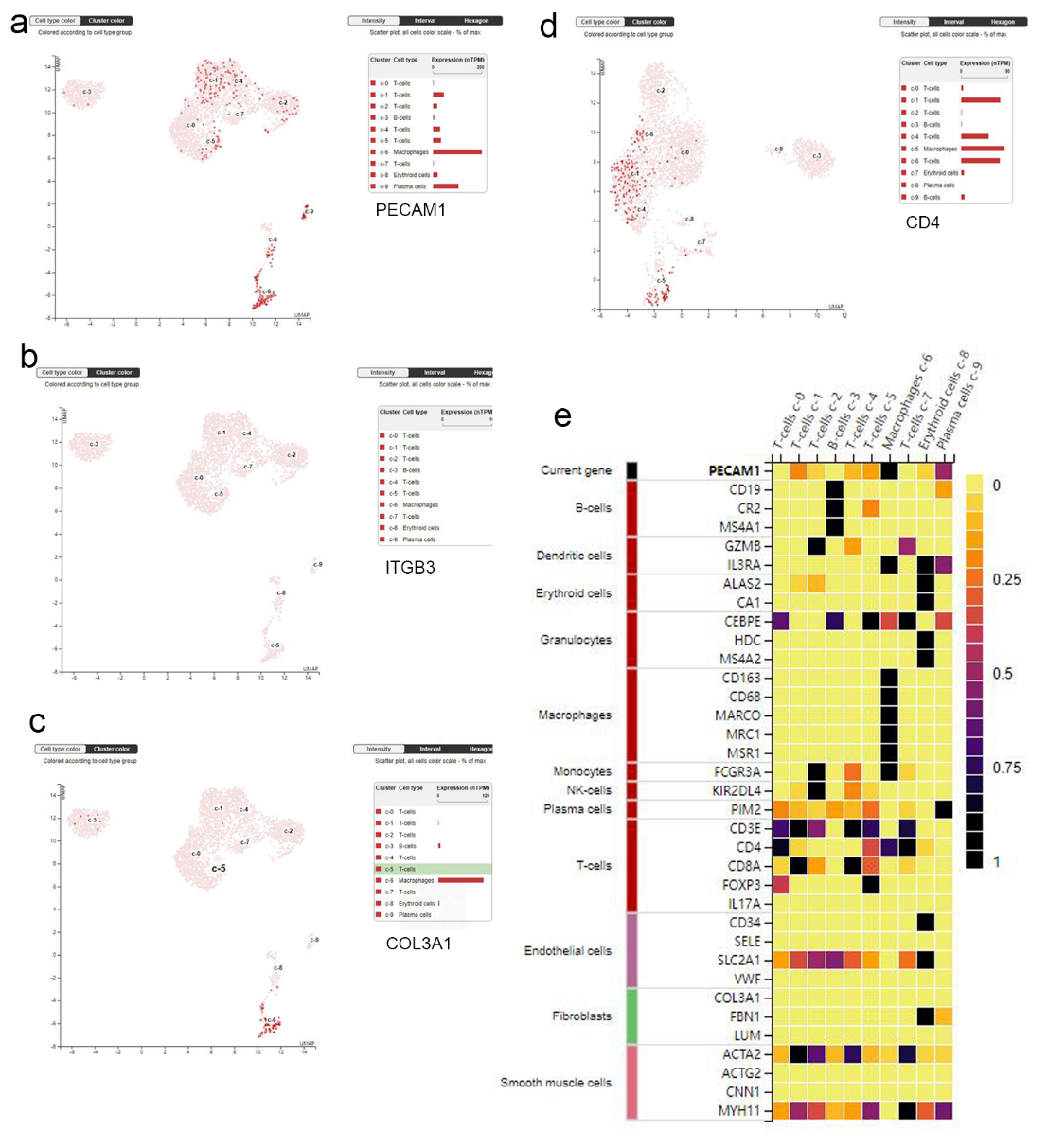


(a-d) Single cell type analysis in THPA database showed high level of PECAM1, CD4 and COL3A1 genes in macrophage. (f) PECAM1, CD4 and COL3A1 had certain cell specificity, and they could be used as marker genes of macrophages.
