# Supplementary material for: PECAM1 plays a role in the pathogenesis and treatment of bone metastases
Source: Front Genet. 2023 Mar 15;14:1151651. doi: 10.3389/fgene.2023.1151651 (PMC10050551; doi:10.3389/fgene.2023.1151651)
Supplement: Supplementary file 1 [file Presentation1.zip › supplementary/Supplementary table 3.docx]

Supplementary table 1: Clinical data of 12 CRC patients and 8 control patients.

| **Age** | **Gender** | **BMI** | **Bone Metastases** | **Other metastases** | | **Primary focus** | | **TNM** |
| --- | --- | --- | --- | --- | --- | --- | --- | --- |
| 49 | Male | 19.7 | YES | | YES | | Lung cancer | T3N2M1 |
| 57 | Male | 26.8 | YES | | YES | | Lung cancer | T3N3M1 |
| 67 | Female | 24.5 | YES | | YES | | Breast cancer | T3N1M1 |
| 70 | Male | 22.7 | YES | | YES | | Prostate cancer | T3N2M1 |
| 49 | Male | 20.1 | YES | | YES | | Liver cancer | T3N3M1 |
| 61 | Male | 26,9 | NO | | NO | |  | / |
| 56 | Male | 22.1 | NO | | NO | |  | / |
| 58 | Female | 28,3 | NO | | NO | |  | / |
| 68 | Female | 23,5 | NO | | NO | |  | / |
| 62 | Male | 25.6 | NO | | NO | |  | / |
| 44 | Female | 19.4 | NO | | NO | |  | / |
| 42 | Male | 20.1 | NO | | NO | |  | / |
